# How I do it — focused Sylvian approach for clipping of middle cerebral artery aneurysms

**DOI:** 10.1007/s00701-025-06423-9

**Published:** 2025-01-10

**Authors:** Milad Neyazi, Rajiv Kumar Khajuria, Sajjad Muhammad

**Affiliations:** Department of Neurosurgery, Medical Faculty and University Hospital Düsseldorf, Heinrich Heine University Düsseldorf, Moorenstr. 5, 40225 Düsseldorf, Germany

**Keywords:** Pterional craniotomy, Intracranial aneurysm, Middle cerebral artery, Minimal invasive surgery, Neurovascular surgery techniques

## Abstract

**Background:**

The Focused Sylvian Approach (FSA) is a refined, minimally invasive technique for clipping small to medium-sized middle cerebral artery (MCA) aneurysms, prioritizing safety and aesthetics.

**Method:**

The craniotomy remains confined to the superior temporal line, with the incision concealed within the temporal muscle. The Sylvian fissure is carefully dissected to preserve venous structures.

**Conclusion:**

FSA achieves optimal vascular control with superior cosmetic outcomes while maintaining adequate exposure for safe aneurysm clipping.

**Supplementary Information:**

The online version contains supplementary material available at 10.1007/s00701-025-06423-9.

## Relevant surgical anatomy

**Fig. 1:**
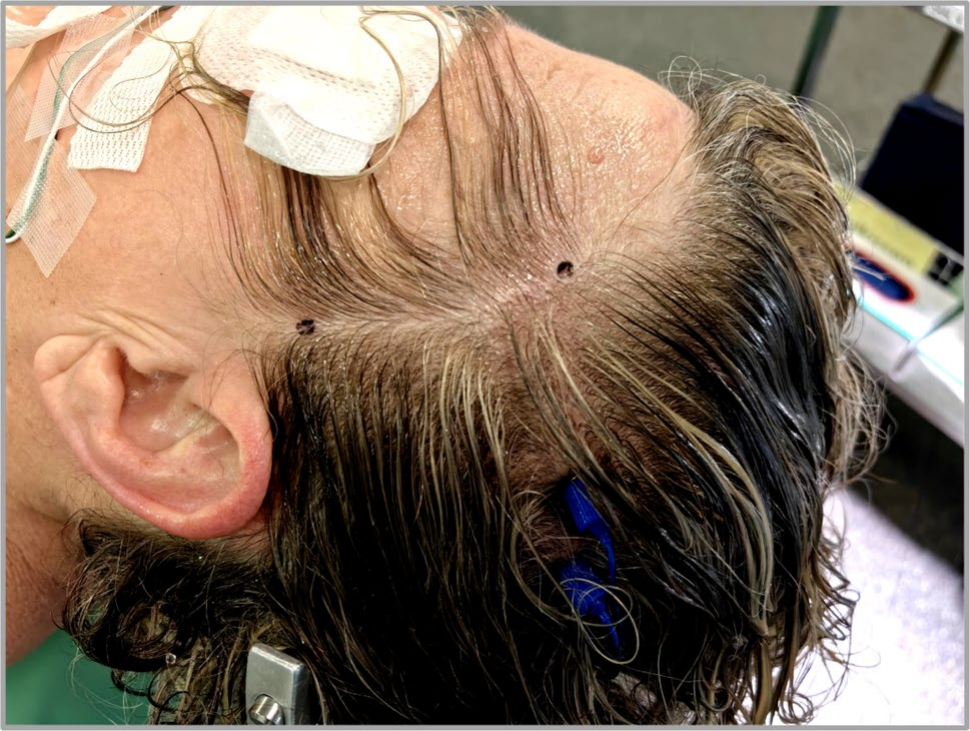
Patient positioning and planned incision showing the 45° head tilt within the Mayfield clamp, with incision concealed behind the hairline

The incision is planned using external anatomical landmarks, including the zygomatic arch, external acoustic meatus, and superior temporal line. The curved incision begins approximately 1 cm anterior to the tragus and 3 cm above the zygomatic arch, staying within the hairline (Fig. 1). This avoids damage to the frontotemporal branch of the facial nerve.

## Description of the technique

### Positioning and craniotomy

**Fig. 2:**
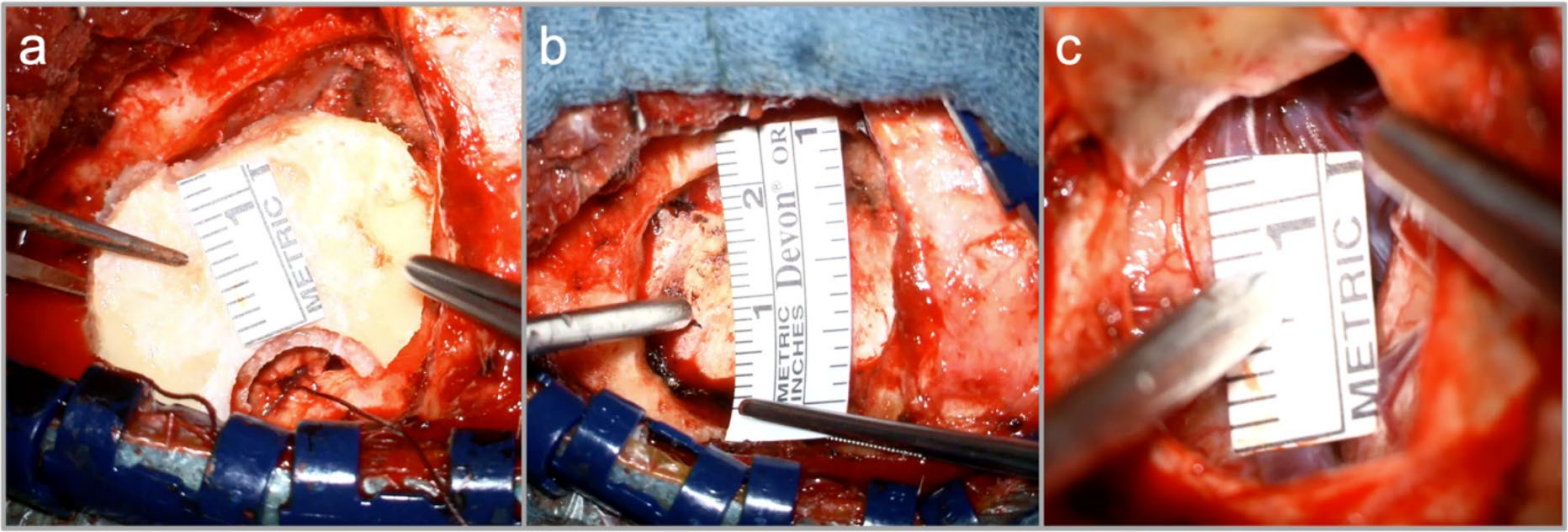
Intraoperative views demonstrating the dimensions of the Focused Sylvian Approach: **a** bone flap, **b** craniotomy margins, and **c** Sylvian fissure exposition

The patient’s head is fixed in a Mayfield clamp and rotated 45° (Fig. 1). The incision follows a gentle curve along the hairline, approximately 6 cm in length. Two-layer dissection proceeds until the fat pad. An L-shaped incision is made in the temporal muscle, which is mobilized anteriorly to expose the pterional cranium while preserving the temporal muscle insertion. A single burr hole is placed cranio-posteriorly, and a 3-cm craniotomy is performed, centered over the Sylvian fissure. The sphenoid ridge is drilled minimally, and the dura is opened towards the ridge with tenting sutures applied to optimize exposure (Fig. 2).

### Sylvian fissure dissection

Using a microscope, the Sylvian fissure is opened distally to proximally. Venous structures are preserved, and cerebrospinal fluid is drained from the Sylvian cistern. The dissection is performed along the frontal side of the superficial Sylvian vein using hydrodissection and bipolar forceps.

### Aneurysm dissection and clip placement

**Fig. 3:**
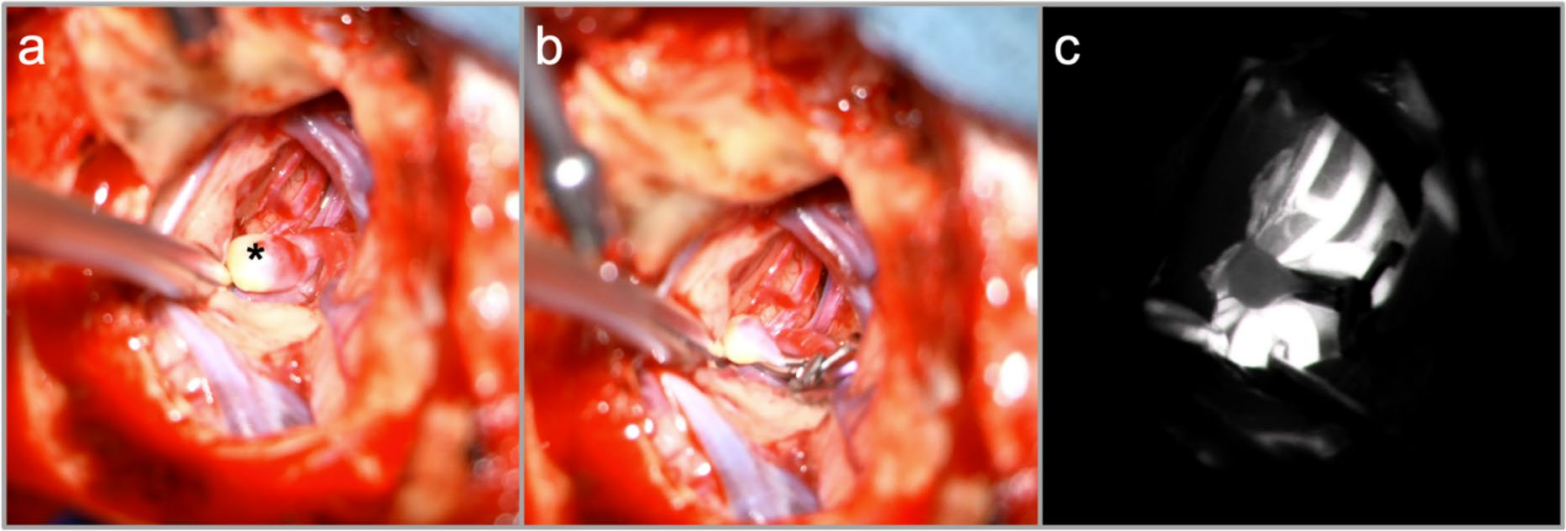
**a** Dissection of the Sylvian fissure and exposition of the M2 bifurcation aneurysm (*) **b** clip ligation of the aneurysm **c** confirmation by intraoperative indocyanine green angiography

Following dissection of the M2 and distal M1 segments, the aneurysm dome is exposed. Temporary clipping of the M1 is employed only if required. The appropriate permanent clip is selected and applied to ensure patency of the MCA bifurcation and temporal M2 branch. Indocyanine green angiography confirms successful aneurysm occlusion and vessel patency (Fig. 3).

### Postoperative management

**Fig. 4:**
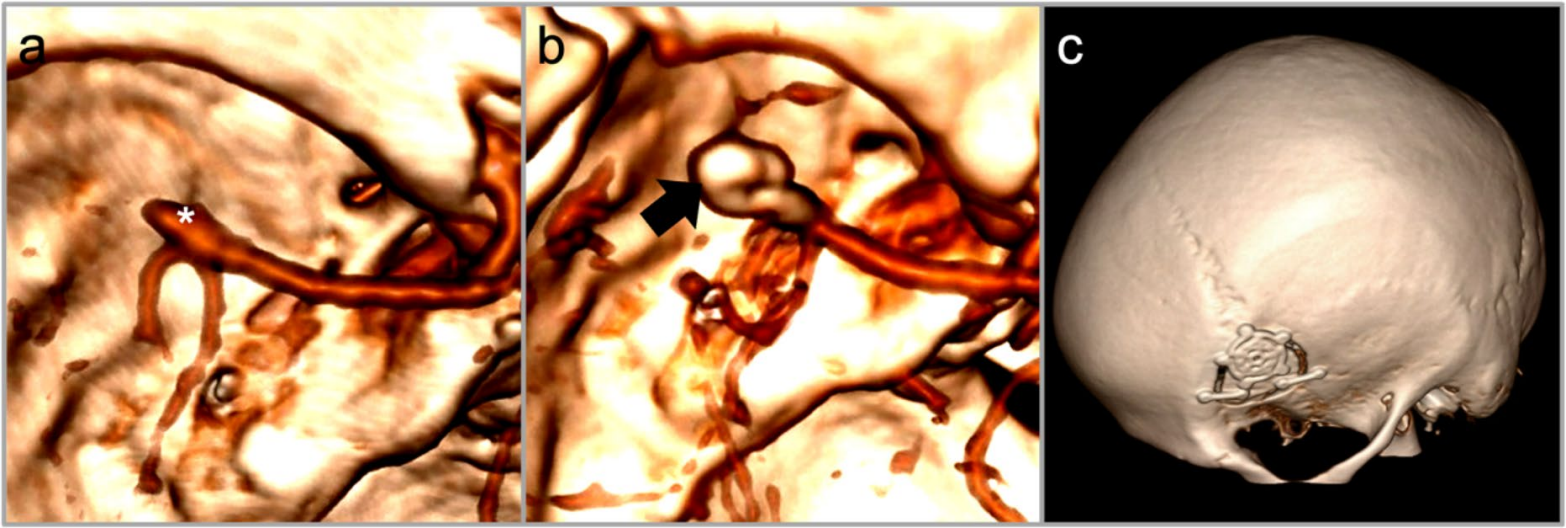
**a** 3D reconstructed CT angiogram of the M2 bifurcation aneurysm (*) pre-operatively and **b** post-operatively following clip ligation (→) **c** post-operative 3D reconstruction demonstrating the relative extent of the craniotomy

Patients are monitored postoperatively in the PACU and transitioned to the regular ward once stable. Regular neurological assessments (GCS) are conducted hourly. A cranial CT combined with CT angiography is performed 6 h postoperatively to evaluate the surgical site and vascular patency (Fig. 4).

### Indications

The FSA is ideal for small to medium-sized incidental unruptured MCA aneurysms (< 10 mm). It provides a minimally invasive alternative to standard approaches, offering safe, adequate exposure and superior cosmetic results.

## Limitations

The reduced size of the craniotomy limits instrument maneuverability. Proper head rotation is critical to avoid conflicts with craniotomy edges. Intraoperative rupture may pose challenges in accessing the surgical corridor, though this can usually be managed with a single suction device. This approach is contraindicated in cases of ruptured aneurysms, as adequate brain relaxation cannot be achieved without CSF drainage from proximal cisterns.

## How to avoid complications

Preoperative angiographic imaging with 3D reconstruction is essential for understanding aneurysm orientation and tailoring the craniotomy. Precise positioning and avoidance of excessive sphenoid drilling minimize risks.

## Specific information for the patient

The FSA offers improved cosmetic outcomes compared to standard approaches, with reduced scar visibility and milder postoperative discomfort. The risks of mastication problems and headaches are minimized due to muscle preservation. Alternative approaches can be discussed based on individual anatomy and surgeon expertise.

## Key points

1. **Indication**: The FSA is optimal for small-to-medium unruptured MCA aneurysms (< 10 mm).
2. **Contraindications**: Not recommended for ruptured aneurysms due to limited ability for proximal cisternal CSF drainage.
3. **Patient Positioning**: The head is fixed in a Mayfield clamp, rotated 45° with a slight tilt.
4. **Incision**: A 6-cm hairline incision, anterior to the tragus, avoids the frontotemporal branch of the facial nerve.
5. **Dissection**: Two-layer soft tissue dissection: epifascial to the fat pad, L-shaped temporal muscle incision mobilized anteriorly.
6. **Craniotomy**: Single burr hole posteriorly, a 3-cm craniotomy confined to the superior temporal line.
7. **Sylvian Dissection**: Distal-to-proximal opening of the Sylvian fissure while preserving venous structures.
8. **Aneurysm Handling**: Temporary M1 clipping if needed; permanent clip placement confirmed by ICG angiography.
9. **Postoperative Care**: PACU observation, regular ward transfer with hourly GCS checks; 6-h CT/CTA for evaluation.
10. **Advantages**: Minimally invasive with superior cosmetic outcomes and sufficient surgical exposure.

## Supplementary Information

Below is the link to the electronic supplementary material.

Supplementary file1 (MP4 139371 KB)

## Data Availability

No datasets were generated or analysed during the current study.

## Abbreviations

FSA: Focused Sylvian Approach
MCA: Middle Cerebral Artery
M1: Sphenoidal Segment of the Middle Cerebral Artery
M2: Insular Segment of the Middle Cerebral Artery

## Declarations

### Use of artificial intelligence

In the preparation of this manuscript, artificial intelligence (AI) tools, specifically OpenAI’s ChatGPT, were utilized for grammar refinement and typographical error correction. These tools were employed solely to enhance the clarity and readability of the text, without altering the scientific content or interpretation of the findings. All intellectual and conceptual contributions remain the responsibility of the authors.
